# HEARING LOSS AND HOSPITALIZATION IN OLDER ADULTS: EVIDENCE FROM THE ATHEROSCLEROSIS RISK IN COMMUNITIES STUDY

**DOI:** 10.1093/geroni/igad104.3387

**Published:** 2023-12-21

**Authors:** Wuyang Zhang, Emmanuel Garcia Morales, Nicholas Reed

**Affiliations:** Johns Hopkins Bloomberg School of Public Health, Baltimore, Maryland, United States; Johns Hopkins University, Baltimore, Maryland, United States; Johns Hopkins Bloomberg School of Public Health, Baltimore, Maryland, United States

## Abstract

Hearing loss is highly prevalent among older adults and is associated with increased healthcare utilization. However, the majority of evidence relies upon self-report measures of hearing and hospitalization which are subjective to validity concerns. Using cross-sectional data from the Atherosclerosis Risk in Communities Study and linked Centers for Medicare and Medicaid Services (CMS) records (2016), we aim to investigate the association between objectively measured hearing loss and record-based hospitalization in a population-based sample. Among 1,330 older adults (Mean age: 79.3 years; Female: 57.8%; Black: 17.4%), hearing loss was measured using pure-tone audiometry and hospitalization outcomes, including occurrence of any hospital stays (yes/no); number of hospital stays; and duration of hospital stays, were ascertained from CMS records. In models adjusted for demographic, socioeconomic, and health characteristics, those with mild and moderate/greater hearing loss had 1.71 (95% CI: 1.13-2.58) and 1.87 (95% CI: 1.18-2.98) times higher prevalence of any hospital stay over 12 months; 2.23 (95% CI: 1.47-3.37) and 1.68 (95% CI: 1.04-2.71) times higher Incident Rate of multiple hospital stays, relative to those without hearing loss. Moreover, both groups had significantly longer durations of hospital stay (mild: Incident Rate Ratio [IRR]=2.41; 95% CI: 1.46-3.98; moderate/greater: IRR=2.32; 95% CI: 1.32-4.07). Using criterion exposure and outcome measures, these findings confirm previous work and provide the first insight into the association of hearing loss with increased inpatient service use. Future work should focus on accommodations for hearing to reduce healthcare utilization and design equitable healthcare systems that meet the needs of all older adults.

